# Phase Transition of High-Surface-Area Glycol–Thermal Synthesized Lanthanum Manganite

**DOI:** 10.3390/ma16031274

**Published:** 2023-02-02

**Authors:** Victor O. Anyanwu, Holger B. Friedrich, Abdul S. Mahomed, Sooboo Singh, Thomas Moyo

**Affiliations:** School of Chemistry and Physics, Westville Campus, University of KwaZulu-Natal, Durban 4000, South Africa

**Keywords:** perovskite, lanthanum, manganese, glycol–thermal synthesis, resistivity, DFT–PBEsol

## Abstract

Cubic and rhombohedral phases of lanthanum manganite were synthesized in a high-pressure reactor. A mixture of La and Mn nitrates with ethylene glycol at a synthesis temperature of 200 °C and a calcination temperature of up to 1000 °C, resulted in a single-phase perovskite, LaMnO_3_ validated using X-ray diffraction. Significant changes in unit cell volumes from 58 to 353 Å^3^ were observed associated with structural transformation from the cubic to the rhombohedral phase. This was confirmed using structure calculations and resistivity measurements. Transmission electron microscopy analyses showed small particle sizes of approximately 19, 39, 45, and 90 nm (depending on calcination temperature), no agglomeration, and good crystallinity. The particle characteristics, high purity, and high surface area (up to 33.1 m^2^/g) of the material owed to the inherent PAAR reactor pressure, are suitable for important technological applications, that include the synthesis of perovskite oxides. Characteristics of the synthesized LaMnO_3_ at different calcination temperatures are compared, and first-principles calculations suggest a geometric optimization of the cubic and rhombohedral perovskite structures.

## 1. Introduction

Nanomaterials of perovskite oxide based on the ABO_3_ system, where the lanthanide occupies the A-site, and an alkaline earth metal or transition metal the B-site, have recently gained popularity and have become a subject of interest for researchers due to the possibility of several applications [1]. For an ideal cubic perovskite structure using the ABO_3_ chemical formula, twelve and six oxygen atoms are bound to the A-site and B-site cations, respectively. These A- and B-sites can accommodate elements with ionic radii that can fit the perovskite structure allowing for precise development of the mixed oxide material [2]. The coordination of the cation and the anion contributes to the high stability of the perovskite structure and substituting either or both sites can influence the use of perovskite-type oxides in electrocatalysis, thermocatalysis, photocatalysis, and energy storage [3,4]. Detailed understanding of the structure and distribution of defects in the perovskite-type oxide are the basis to elucidate oxygen ion transport in the pure phase material. Among structural defects present in perovskite-type oxides, the Jahn–Teller (JT) effect describes the geometrical distortion of B-site cations in perovskite oxides. In a neutron diffraction study, Alonso et al. [5] studied the evolution of the JT effect in RMnO_3_ (R = rare earth) powders prepared from citrate precursors. They concluded that the JT distortion mediates the elongation of the axial bonds of the thermally treated perovskite, which further results in the occurrence of different cationic–anionic bond lengths.

Perovskite oxide catalysts used in oxidative catalysis are just as good as widely used metal oxides, such as CeO_2_ and precious metals, such as Pt and Pd [1]. The application of the ABO_3_ system in oxidative catalysis showed that their performance is due to the presence of active peroxide species activated by lattice oxygen and considerably reduced activation energy barriers [6]. With a host of alkaline earth metals being employed in the catalytically active B-site of the perovskite group of materials, manganese-based perovskite catalysts have been shown to be efficient, particularly in automotive exhaust catalytic converters, when compared to other mixed oxide systems [7]. The catalytic performance of the Mn-based system is also influenced by the ability of the perovskite to accommodate oxidative non-stoichiometry [8]. Apart from the known underlying principles, such as the partial substitution of the B-site where the Mn cations reside, Mn oxidation states positively contribute to the redox property of the perovskite [9,10]. Furthermore, the nanoscale crystals of Mn-based perovskites often exhibit properties different from their bulk counterparts [11]. This difference was observed in studies comparing the specific surface area of the bulk material with nanoscale Mn perovskite, reported by Kulandaivelu et al. and Zhong et al. [12,13]. They suggested that the decrease in grain size resulting in a larger surface area of the nanoscale perovskite led to a decrease in the phase transition temperature of perovskites. In addition, due to various favourable properties of the Mn perovskite-type oxides, the perovskite material can substitute noble metal catalysts in heterogeneous catalytic reactions [1]. Such perovskite-structured materials can exist in cubic, orthorhombic, and rhombohedral phases [14,15]. The rhombohedral phase, prepared via high thermal treatment, contains excess oxygen caused by cationic vacancies [16,17]. Rodriguez-Carvajal et al. [15] studied the defectiveness of LaMnO_3_ and concluded that the presence of Mn^4+^ resulted in the creation of cationic vacancies at the site containing the lanthanum cation, and in the B-site containing the Mn cation. However, Wang et al. [18] showed that different calcination temperatures influenced the crystal phases of the lanthanum–strontium–manganite material prepared to investigate its electrocatalytic behaviour towards the oxygen reduction reaction (ORR). There were attempts to determine which perovskite phase was more active, including the work by Ashok et al. [19], who reported a slight elongation of the B-site and O–anion bond in the cubic structure, which led to favourable oxygen chemisorption that facilitated bifunctionality towards the ORR. In addition, LaMnO_3_ with a rhombohedral structure showed reasonable performance for use in the catalytic oxidation of CO, the selective oxidation (SELOX) of CO, oxygen reduction reactions (ORR), and oxygen evolution reactions (OER) [9,18].

The lattice structure of LaMnO_3_ perovskite exists as an ideal cubic Pm-3m space group at room temperature. Due to the appearance of cooperative rotations of the MnO_6_ oxygen octahedral, the lattice deviates from this ideal structure. A temperature change generally induces LaMnO_3_ structural phase transition. According to Illiev et al., LaMnO_3_ heat-treated at 900 °C, showed the orthorhombic Pbnm space group [14]. However, when Qiu et al. and Norby et al. treated LaMnO_3_ at a temperature > 900 °C, the rhombohedral space group R3$\bar{c}$ was identified [20,21]. The synthesis method and calcination temperature have been shown to influence the perovskite lattice structure.

Various synthesis methods for preparing perovskites with a variety of rare earth elements occupying the A-site have been reported [22,23,24]. The citrate method was used in the preparation of samarium and neodymium compounds which were compared with LaFeO_3_ for methane combustion. The order of activity towards methane combustion was reported as La > Nd > Sm [3]. Apart from the citrate method, other methods, such as reactive mechanical milling [25], solid-state reactions [26,27], sol–gel [28,29], co-precipitation [30], thin-film deposition [31], single-crystal growth [15], and solution combustion [32] have produced materials with various physicochemical properties. Another method for bulk production of the perovskite materials is using the glycol–thermal technique, which has been applied successfully to obtain different types of metal-oxides, spinels, and perovskite-type oxides [33,34]. This method was effective in the synthesis of a structured manganese spinel via the activation of chloride compounds that favoured the single-phase formation of uniform, nanocrystalline, and non-agglomerated materials [33]. A report by Tomaszewski et al. [35] describes the use of a microwave-assisted glycol–thermal method to prepare different La-based nano-crystalline perovskite oxides.

This study investigates a method of synthesizing perovskites containing La and Mn with controlled grain size with no use of a chelating agent and seeks to determine the relationship between the synthesis procedure and the crystal structure transition, morphology, texture, and particle size. For the first time the glycol–thermal method was used to synthesize high-surface-area LaMnO_3_ perovskite powders which was calcined from 700 to 1000 °C. The perovskite oxides demonstrated a phase transformation from the cubic Pm-3m to the R$\bar{3}$c crystal system in the temperature range of 800–900 °C. Theoretical evaluation of the transition of the LaMnO_3_ perovskite structure using first-principles calculations was used to support experimental results.

## 2. Experimental

### 2.1. Synthesis and Structural Calculations

LaMnO_3_ perovskite oxides were prepared using the glycol–thermal synthesis method following a procedure reported previously [36]. A solution of lanthanum and manganese was prepared by dissolving lanthanum nitrate (62.6 wt.%) and manganese nitrate (37.4 wt.%) in 500 mL of deionized water. Ammonia was added dropwise to the La/Mn solution to increase and maintain the pH at 9 to facilitate precipitation. The mixture was stirred continuously to allow for complete precipitation. The precipitate was filtered and washed several times with deionized water, until a pH of 7 was obtained. Excess water was further removed with a final wash with ethanol. The precipitated gel was placed in a 600 mL glass liner, to which 200 mL of ethylene glycol was added and vigorously stirred to obtain homogeneity. The vessel was placed in a PARR reactor (Moline, IL, USA), set at a reaction temperature of 200 °C and stirred at 300 rpm for 6 h. The resulting gel was transferred from the glass liner after the reaction came to completion, and washed using deionized water and ethanol to remove any trace of ethylene glycol. The gel was dried using a 200 W IR lamp for 12 h. The resulting solid was finely crushed using a mortar and pestle and finally calcined at 700, 800, 900, and 1000 °C for 6 h to obtain materials denoted as LM 700, LM 800, LM 900, and LM 1000, respectively.

### 2.2. Density Functional Theory Calculations

Density functional theory (DFT) theoretical calculations were executed using Biovia material studio software 2017 v17.1.0.48, with the CASTEP (Cambridge sequential total energy package) geometry optimization module. The 3D structures of the LaMnO_3_ cubic and rhombohedral symmetry used for the CASTEP calculations are shown in Figure S1, supplementary information. A plane-wave energy cut-off of 450, 650, and 800 eV and a very close Monkhorst–Pack k-point grid were applied to converge the electron system. LaMnO_3_ density of states (DOS) and band structure of the cells as a function of energy were compared. Pseudo atomic calculations performed for La (5s^2^, 5p^6^, 5d^1^, and 6s^2^), Mn (3d^5^ and 4s^2^), and O (2s^2^ and 2p^4^) converged successfully for all plane-wave energy cut-offs. These sets of parameters were sufficient to produce the total energy convergence, BFGS maximum enthalpy (eV), frequency (cm^−1^), modulus/stress (GPa), maximum force, and maximum displacement (Å) obtained. In the configuration setup, the “spin polarized” option was chosen, which ensured that spin–orbit interaction in the calculation was obtained.

### 2.3. Material Characterization

#### 2.3.1. Phase Identification

X-ray diffraction (XRD) of the powders was used to determine the phase transformation during the calcination process, using a BRUKER AXS (Karlsruhe, Germany) multipurpose D8-Advance X-ray diffractometer. Diffraction parameters were a 2*θ* range of 20 to 90°, with a step size of 0.034°. A Cu–Kα (λ = 1.5406Å) radiation source was used in all experiments. Rietveld refinement analysis of the XRD data was performed to determine the lattice of the perovskite oxide structure.

#### 2.3.2. Thermogravimetric Analysis

Thermogravimetric analysis was carried out using a Perkin Elmer (Waltham, MA, USA) simultaneous thermal analysis STA 6000 instrument, using a ramp heating rate of 10 °C/min. The thermogravimetric analysis was performed over a temperature range from 25 to 1000 °C.

#### 2.3.3. Infrared Spectroscopy

To compare the crystalline phases in the samples, Fourier transform infrared (FTIR) spectroscopy data were collected. Infrared spectra of the LaMnO_3_ materials were recorded using a Perkin Elmer (Waltham, MA, USA) spectrometer in universal attenuated total reflectance (ATR) mode.

#### 2.3.4. Raman Spectroscopy

Raman spectroscopic data were obtained with a Renishaw (New Mills, UK) inVia Raman Spectrometer. The spectra were obtained using an excitation wavelength of 514 nm and the material was scanned between 100 and 3000 cm^−1^.

#### 2.3.5. BET Surface Area

The BET surface areas of the samples were determined using a Micromeritics (Norcross, GA, USA) TriStar II instrument. The powder samples were dried and degassed by heating gently to 90 °C for 1 h, then at 200 °C under a flow of N_2_ for 3 h, using a Micromeritics (Norcross, GA, USA) FlowPep 060 instrument, prior to analysis.

#### 2.3.6. Electron Microscopy

For scanning electron microscopy (SEM), the samples were fixed onto a carbon tape and coated with gold to prevent charging during analysis. SEM and energy-dispersive X-ray spectroscopy (EDX) were conducted on a ZEISS (Oberkhochen, Germany) FEG–SEM Ultra Plus instrument.

Transmission electron microscopy (TEM) and high resolution (HR-TEM) images were obtained using a Jeol (Tokyo, Japan) JEM-1010 electron microscope. Images were analysed using ImageJ software. Approximately 0.2 mg of the sample was placed in ethanol and sonicated for 20 min. The mixture was placed on a copper grid and dried in air. MegaView III Software Imaging captured TEM and HR-TEM images. Furthermore, i-TEM or ImageJ software were used for image analysis including measuring the particle size.

#### 2.3.7. Electrical Measurement

The main focus of this work is the structural transformation of lanthanum manganite perovskite oxides calcined from 700 to 1000 °C. During this work, it became apparent that measurements of the electrical resistivity at room temperature would assist in understanding the various contributions to the structural changes. At room temperature, the bulk resistivity was evaluated using a Keithley (Cleveland, OH, USA) interactive source meter (SMU) instrument (Model 2450) and a collinear four-point probe method (Jandel model) on freshly pelletized samples. The four-point probe spacing was 1 mm.

## 3. Results and Discussion

### 3.1. Thermal and Structural Analysis

**Figure 1:**
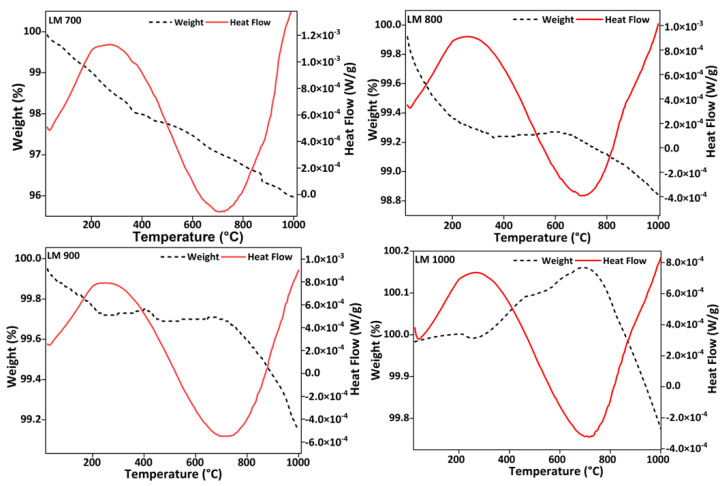
Thermogravimetric profiles of LM 700, LM 800, LM 900, and LM 1000.

The comparative thermogravimetric analytical curves for lanthanum manganites, synthesized using a template-free glycol–thermal method and calcined at different temperatures, are presented in Figure 1. Impurity determination in the LaMnO_3_ powder through TGA conducted at atmospheric pressure showed that some impurity elimination or decomposition took place in the calcined samples. The heat-flow curves show the presence of maxima and minima differential thermal analysis (DTA) exothermic peaks. The maxima were observed at approximately 300 °C and minima at about 700 °C. The total weight loss between 100 and 1000 °C was 3.56 wt.% for the LM 700 powder. The weight loss decreased significantly to 0.74, 0.71, and 0.21 wt.% for the LM 800, LM 900, and LM 1000 samples, respectively. The major weight loss occurred between 100 and 200 °C, corresponding to the loss of physically adsorbed water and ethylene glycol. The second weight loss between 200 and 600 °C corresponds to the loss of the chemically bound hydroxyl groups, whereas the loss of weight above 700 °C is the result of CO_2_ release from the decomposition of carbonate species. The hydroxyl and the carbonate content decreased with increasing calcination temperature. An increase in mass was observed in the LM 1000 sample. The weight gain from the initial weight to the final weight for temperatures up to 860 °C was 0.22 wt.%. This observation can be an effect of oxygen intake of the sample [37].

**Figure 2:**
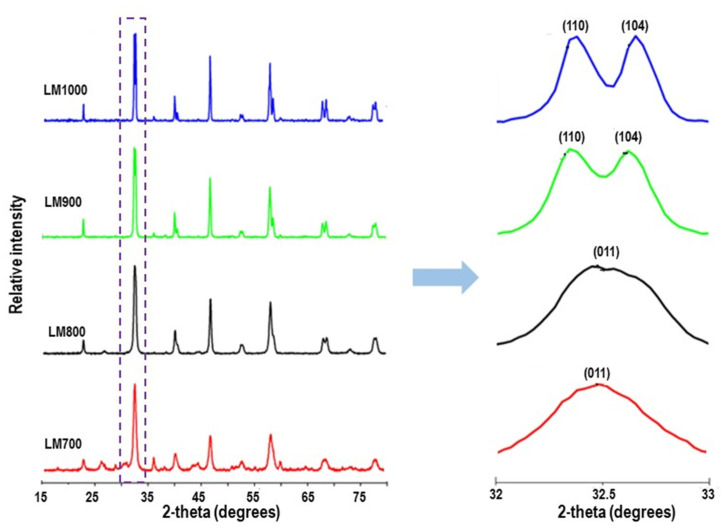
X-ray diffraction patterns of LM 700, LM 800, LM 900, and LM 1000.

Powder XRD patterns for all the samples are shown in Figure 2. The patterns show peaks for lanthanum manganites, Pm-3m, and R$\bar{3}$c structures [LaMnO_3_ (ICSD-01-075-0440, 01-082-1152)], captured with peaks located at 2*θ* values of 22.90°, 32. 61°, 40.22°, 46.82°, 58.18°, 68.39°, and 77.86°. The crystallinity of the compounds increased with calcination temperature and the XRD patterns are characteristic for La-based perovskites showing clear, sharp, and well-defined reflections. For LM 700, the unidentified impurity peaks at 21, 36, and 60° 2*θ* were below and above the perovskite reflection line (110), while the major perovskite lines remained unaffected. The cubic LaMnO_3_ symmetry, resulting from reducing conditions during synthesis, was visible in LM 700 and LM 800. At a thermal treatment of 800 °C, the low intensity impurity peaks present in the LM 700 sample diminished. Peak profile sharpening, due to crystallite size increase and decrease in lattice strain was observed as the calcination temperature increased [38]. The unit cell of the ideal cubic phase observed in LM 700, indicated by comparison of the axes, transformed to a doublet in LM 1000. The unit cell dimensions listed in Table 1 correlate with the data published in a recent experimental and theoretical paper [39]. Prior to the Rietveld refinement analysis of LM 1000, we observed that the initial cell volume of LM 900 decreased by 0.6 Å^3^**,** which can be attributed to a minor error that could arise from using unrefined values to calculate cell volume.

**Figure 3:**
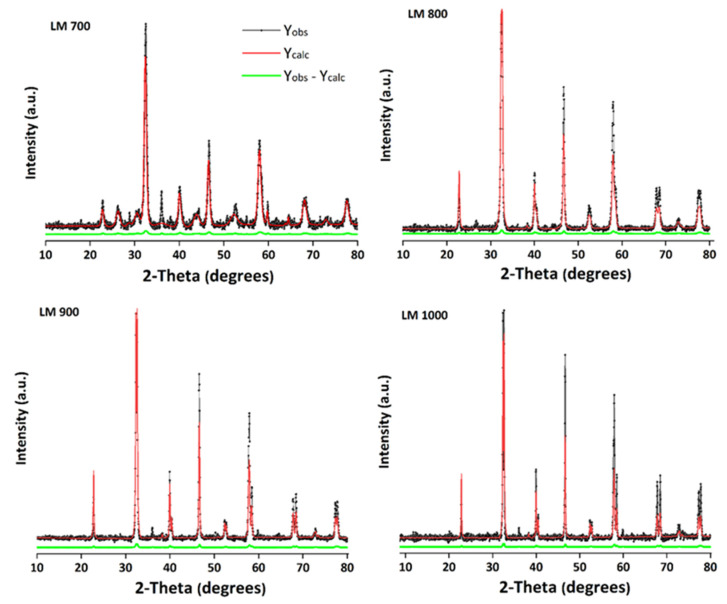
The Rietveld refinement plots for LM 700, LM 800, LM 900, and LM 1000.

Huang et al. [40] emphasized that phase transition of the lanthanum-based perovskite from the orthorhombic phase to the rhombohedral phase can occur through thermal treatment. This was verified using a neutron powder diffraction experiment. Rhombohedral symmetry, observed in the higher 2*θ* values of the LM 900 and LM 1000 samples was associated with the increase in calcination temperature. The phase change, quantitatively refined as a function of the calcination temperature, carried out using Xpert High score (Figure 3, Table 1) confirmed a minimal increase in the unit cell volume. This account agrees with a report by Wei et al. [41], who observed an increase in unit cell volume directly proportional to the grain sizes. For the Rietveld refinement, to obtain 100% LaMnO_3_, parameters reported by Norby et al. [20] and Huang et al. [40], such as the atomic coordinates, isotropic temperature factors, *B*, and population factor (excluding Mn) were adopted. Refinement using the pseudo-Voigt function allowed for thirteen structural and ten profile parameters. Table 1 shows the refinement parameters and data obtained from the refinement. Plots of Y_(obs)_, Y_(cal)_, and Y_(obs-cal)_ are shown in Figure 3.

Single phases from refinement were observed, suggesting that the samples were pure and Mn occupied sites in the perovskite structure. The refinement of the cubic phase indicated no distortion of the unit cell, while the rhombohedral structure showed a possible distortion of the MnO_6_ octahedron. From evidence in refinement of the LM 700 and LM 800 samples, the 180° Mn-O-Mn angle of the cubic structure deviated from cubic to an irregularly hexagonal structure, thereby lowering the symmetry. As Jahn–Teller distortion leads to lower MnO_6_ symmetry, the cubic structure easily transforms to a rhombohedral phase. The results obtained from the Williamson–Hall plot and Rietveld refinement (Table 2), which indicated changes in bond lengths, suggested the presence of defects in the perovskites with La vacancies and the presence of Mn^3+^ and Mn^4+^.

**Figure 4:**
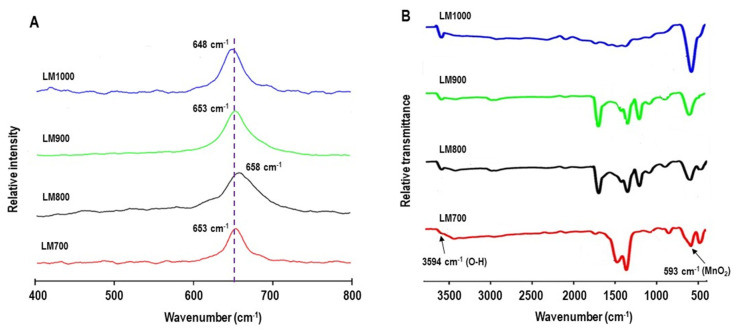
(**A**) Raman and (**B**) infrared spectra of the samples.

Raman spectra confirmed the crystallinity and pure phases of the LaMnO_3_ calcined at different temperatures (Figure 4A). In each individual spectrum, very intense peaks at 648, 653, 658, and 653 cm^−1^ are associated with the B_2g_ mode and were markedly consistent. However, the line feature that depicts the A_g_ mode, which is linked to Jahn–Teller distortion, was absent. This is probably due to a quantified single phase present in the materials, shown clearly byXRD analysis.

Infrared spectroscopy was performed to investigate the impurity content and chemical bonding states between the lanthanum-oxygen and manganese-oxygen atoms in LaMnO_3_ samples that produced different structures at different calcination temperatures (Figure 4B). All absorption peaks observed confirmed the polycrystallinity of the LaMnO_3_ samples. Visible bands observed around 3500–3600 cm^−^^1^ and 500–600 cm^−^^1^ correspond to the bond stretching vibrations of hydroxyl group (V_O-H_) and the M–O bond (V_M-O_) stretching vibrational modes, respectively, which suggest the formation of a metal perovskite oxide framework [42,43].

### 3.2. Textural Properties

Table 3 shows a summary of the textural properties of the LaMnO_3_ materials, which includes the specific surface area, pore volume, and average pore size. The pore volume and average pore size were obtained from the desorption branch of the respective N_2_ isotherm using the Barrett–Joyner–Halenda (BJH) method. Surface area, pore volume, and average pore size decreased with an increase in calcination temperature. The surface areas of the LaMnO_3_ perovskite prepared using the glycol–thermal synthesis route were 33.1, 14.6, 6.6, and 2.2 m^2^/g for LM 700, LM 800, LM 900, and LM 1000, respectively. Using the sol–gel combustion route, Sui et al. [44] produced LaMnO_3_ perovskite with a surface area of 5.2 m^2^/g. The particle size of the powders was determined quantitatively using the expression D_BET_ = 6000/ρ (g/cm^3^) × surface area (m^2^/g); the results are shown in Table 2. The calculated particle sizes are consistent with results published by Sui et al. [44].

### 3.3. Electron Microscopy

**Figure 5:**
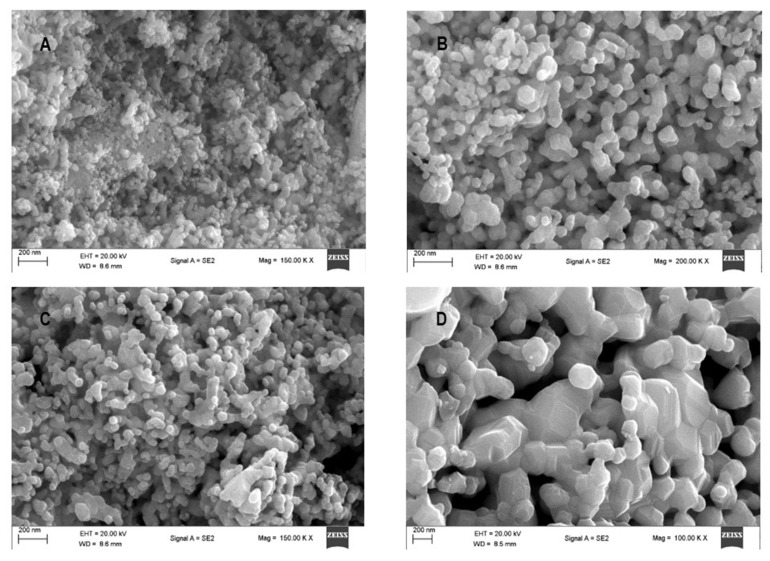
SEM micrographs of LM 700 (**A**), LM 800 (**B**), LM 900 (**C**), and LM 1000 (**D**).

Scanning electron microscopy (SEM) images presented in Figure 5 show perovskite particles with shapes resembling cubes in the samples calcined at 700 and 800 ℃, whereas the dominant shapes in the LM 900 and LM 1000 samples are hexagonal. As shown in Figure 5B, increased particle size and agglomeration contributed to the observed closely packed cube-like LaMnO_3_ particles.

**Figure 6:**
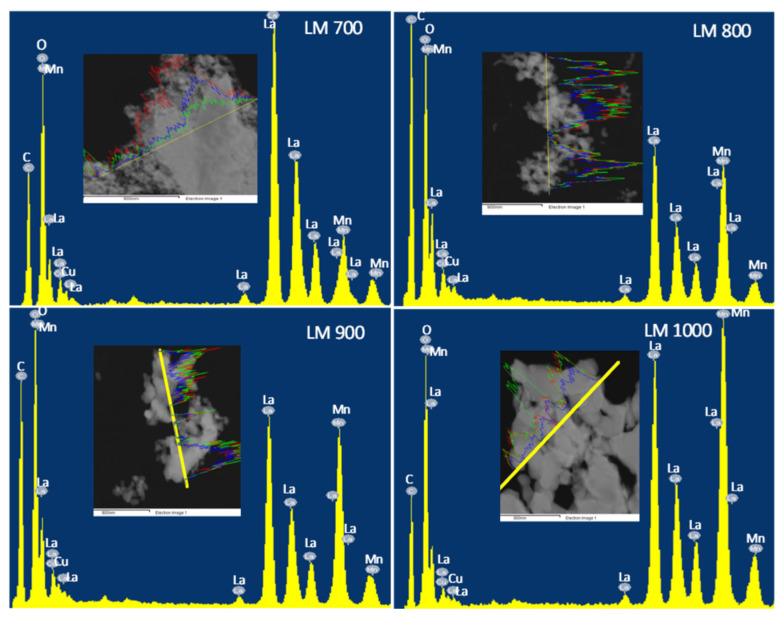
EDX plots of LaMnO_3_ (■ La, ■ Mn, and ■ O).

Energy dispersive X-ray analysis (EDX), Figure 6, shows the presence of all the expected elements in the LaMnO_3_ perovskite. Table 4 gives the respective atomic percentages of the elements in the perovskite samples. In general, EDX results are close to the nominal values, thus confirming the actual composition of the powder samples. The results from electron microscopy images and EDX suggest that the glycol–thermal technique and calcination temperatures influenced the morphology of the LaMnO_3_ samples. Additionally, the lattice d-spacing was in the range of 0.3–0.4 Å, which shows consistency with the experimental results published by Ortiz-Quiñonez et al. [45].

**Figure 7:**
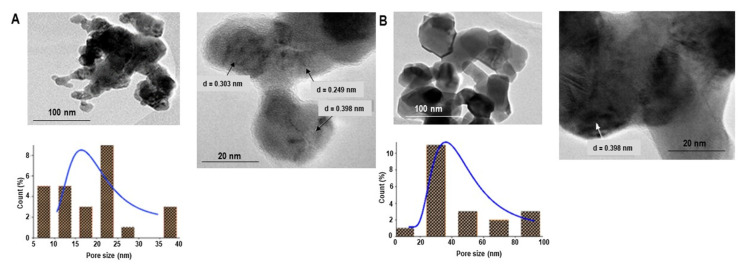
TEM images and particle size histograms of LaMnO_3_ calcined at (**A**) 700 °C and (**B**) 900 °C.

Figure 7 shows transmission electron microscopy (TEM) images of LaMnO_3_ samples calcined at 700 and 900 °C. The images of the other samples are in the supplementary information, Figure S2. The micrographs clearly show the cubic and hexagonal structures, smooth surfaces, uniform sizes, low aggregation, and well-distributed nanoparticles. From TEM analysis, particle size-induced phase transition from the cubic to the hexagonal phase could be observed at about 47 nm, with mean values of 18.7, 38.5, 44.7, and 89.6 nm obtained for the samples calcined at 700 °C, 800 °C, 900 °C, and 1000 °C, respectively. The mean particle size showed the growth of the LaMnO_3_ grains and thus the size-induced phase transition as the calcination temperature increased to 1000 °C. It is interesting that the value of the crystallite size calculated using the Rietveld refinement method was nearly similar to the mean diameter of the particle size calculated using TEM due to the regular shape of the nanoparticles with cubic and hexagonal morphologies, which were observed in the TEM micrograph.

Although most of the reported data for lanthanum manganite shows hexagonal and orthorhombic crystals [15,29,46,47], the TEM images in this study show cubic and rhombohedral crystals with an average lattice spacing of 0.398 nm, corresponding to the (011 and 110) d-spacings of LaMnO_3_.

### 3.4. Electrical Resistivity

The room-temperature resistivities of the LaMnO_3_ samples measured using the four-point probe technique on pelletized samples are shown in Table 5. The values show that the resistivity generally decreased associated with the increased calcination temperature. The change in resistivity was more drastic as the sample phase transformed from the cubic to the rhombohedral structure. The resistivity shows that the LaMnO_3_ perovskite phase transformation observed structurally can also be associated with significant changes in electronic properties.

**Figure 8:**
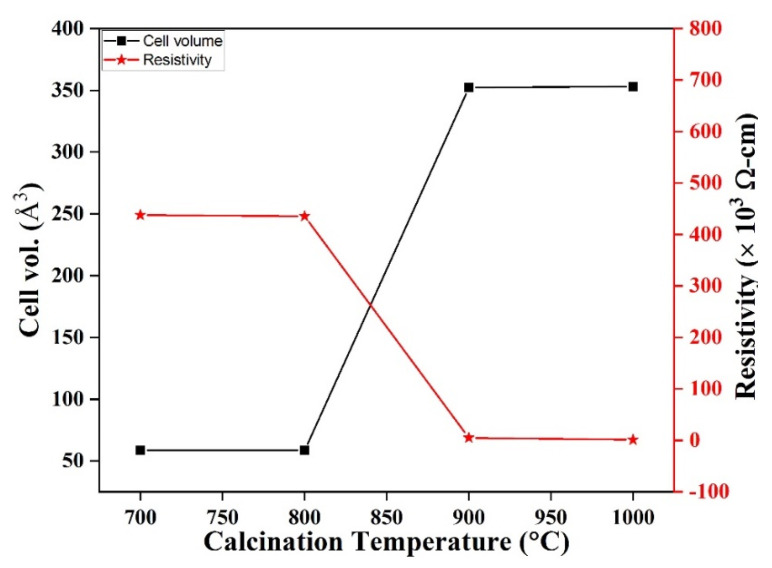
A plot of the cell volume and resistivity of LaMnO_3_ calcined at different temperatures.

Figure 8 shows the cell volume and resistivity of LaMnO_3_ calcined at different temperatures. The plot shows that as the calcination temperature and cell volume increased, the resistivity decreased.

As the calcination temperature tends to influence resistivity it also affects conductivity. The resistivity at room temperature indicates that ***ρ*** decreased from 18.47 MΩ cm at 39 nm to 20.91 K Ω cm for 90 nm due to increased particle size (Table 2). This trend can be attributed to the increasing content of Mn^4+^ ions which contribute to the holes that are produced in the perovskite compounds [48]. Additionally, increased oxygen and cation vacancies, and reduced porosity and grain boundaries can influence the low resistivity values recorded in the compounds [49].

### 3.5. Computational Analyses

DFT calculations can provide an interesting guidance to Rietveld refinement. Rietveld refinement experimental data in combination with CASTEP geometric optimization provides a powerful alternative to standard approaches in cases where the information content of the powder diffraction pattern alone is insufficient to distinguish between different structures. The structural properties of ideal cubic (Pm-3m) and rhombohedral (R$\bar{3}$c) symmetry of LaMnO_3_ were studied. In CASTEP calculations, the structural properties of the perovskite structure were verified in relation to the experimental data, where phase transition was observed. After geometric optimization, the space group obtained correlated with one of the twelve possible groups analysed theoretically for double perovskite [26]. The initial and final total volumes of the lattice after structural optimization; bulk DFT lattice lengths a, b, c; total energy; and stress are shown in Table 6 and Table 7.

**Figure 9:**
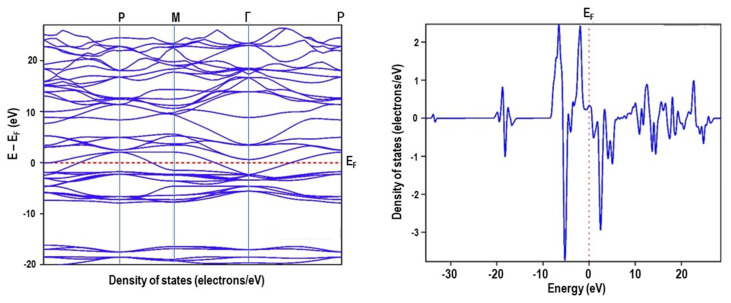
CASTEP GGA–PBE band structure and DOS of cubic perovskite LaMnO_3_.

**Figure 10:**
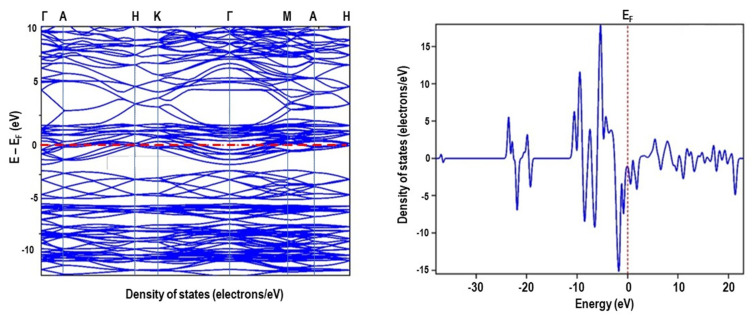
CASTEP GGA–PBE band structure and DOS of rhombohedral perovskite LaMnO_3_.

The band structure calculation was performed after the optimization. A high success level was observed in convergence after a certain number of optimizations using the Broyden–Fletcher–Goldfarb–Shanno (BFGS) algorithm, a second order optimization method. Figure 9 and Figure 10 show the cubic and rhombohedral symmetry band structure and density of states for the spins. The k-points in the desired k-paths followed within the zone boundary along the direction of X → Γ → M → Γ→ P and Γ → A → H → K → Γ→ M→ A→ H for cubic and rhombohedral LaMnO_3_ structures, respectively. For the spin states, the figures show a continuous behaviour of the DOS through the Fermi level which give a metallic characteristic.

The results from the DFT calculations using the GGA–PBEsol module show evidence of the structural stability that is consistent with other computed data of cubic and rhombohedral lanthanum manganite [50,51]. Additionally, from the decrease in the unit cell volume observed at a higher energy cut-off (eV) for the LaMnO_3_ R$\bar{3}$c symmetry, we can attribute this change to an increase in crystallite size, as also observed in our experimental data [52,53].

## 4. Conclusions

Lanthanum manganite, LaMnO_3_, was synthesized using a glycol–thermal synthesis method without the use of a chelating agent. This involved the mixture of La and Mn nitrates which yielded perovskite powder with reduced particle sizes (D_TEM_ = 19, 39, 45, and 90 nm) and high surface areas of 33.1, 14.6, 6.6, and 2.2 m^2^/g for LM 700, LM 800, LM 900, and LM 1000, respectively. Implementing DFT first-principles calculations, a successful analysis of the physical properties of ideal cubic and rhombohedral structure showed the optimized cell structure of LaMnO_3_ perovskite. When compared to the theoretical data, the experimental results showed that the unit cell lattice of the synthesized powder transitioned from the cubic phase to a rhombohedral perovskite structure at a higher thermal treatment with minimal unit cell volume shrinking. The lanthanum manganite powder in the cubic phase showed signs of transitioning to the rhombohedral symmetry in the calcination temperature range of 800–900 °C; then consisting of nanometre-sized particles with a highly crystalline structure. The resistivity of the lanthanum manganite perovskites decreased consistently as the calcination temperature increased. At a calcination temperature of 1000 °C, particles agglomerated and grain size tripled. CASTEP calculations confirmed that the initial unit cell volume for the cubic and rhombohedral symmetry differed slightly from the final unit cell volume. The observation of minimal unit cell volume shrinking is consistent in both the experimental and theoretical data.

## Figures and Tables

**Table 1:** Experimental cell volumes and parameters obtained from Rietveld refinement indicating cubic and rhombohedral phases.

Samples	Refinement				
	Cell Volume (Å^3^)		Lattice Parameters (Å)		
	Initial	Final	A	b	c
LM 700	58.41	58.46	3.881	3.881	3.881
LM 800	58.41	58.53	3.883	3.883	3.883
LM 900	353.8	352.2	5.519	5.519	13.353
LM 1000	353.2	353.0	5.524	5.524	13.358

**Table 2:** Grain and particle size of LaMnO_3_ samples.

Sample	Crystallite Size (nm)	W–H Plot		Rietveld Refinement		D_BET_ (nm)	D_TEM_ (nm)
		Size (nm)	Strain (%)	Size (nm)	Strain (%)		
LM 700	8.1	12.8	0.10	18.9	0.525	28	19
LM 800	10.5	23	0.10	27.4	0.476	61	39
LM 900	12.0	26	0.10	28.4	0.234	141	45
LM 1000	15.2	48	0.02	62.3	0.034	459	90

**Table 3:** N_2_ adsorption studies.

Samples	Surface Area (m^2^/g)	Pore Volume (cm³/g)	Pore Size (Å)
LM 700	33.06	0.2141	115.4
LM 800	14.60	0.0441	143.7
LM 900	6.635	0.0077	146.4
LM 1000	2.195	0.0058	164.0

**Table 4:** Summary of the elemental compositions detected using EDX.

Sample	O		Mn		La	
	wt.%	at.%	wt.%	at.%	wt.%	at.%
LM 700	18.1	55.8	25.2	19.1	50.8	20.5
LM 800	17.9	56.3	12.9	11.5	61.5	22.6
LM 900	18.4	57.3	17.3	15.3	55.4	20.2
LM 1000	14.9	51.0	20.0	20.2	59.5	23.7

**Table 5:** Sheet resistances and resistivities of samples calcined at different temperatures. (The pellets thicknesses were 0.518 mm, 0.531 mm, 0.516 mm, and 0.538 mm for LM 700, LM 800, LM 900, and LM 1000, respectively. The spacing between probes was 1.00 mm).

Samples	Sheet Resistance	*ρ* (kΩ cm)
LM 700	18.64 MΩ	437.7
LM 800	18.47 MΩ	435.3
LM 900	20.91 kΩ	4.890
LM 1000	3.665 kΩ	0.894

**Table 6:** Data of theoretical calculations using the CASTEP module with the Pm-3m space group.

Cut-Off (eV)	Total Energy (eV)			Cell Volume Å^3^	Final Energy (eV)	BFGS Final Enthalpy (×10^3^ eV)	BFGS Bulk Modulus (GPa)	Lattice Parameters (Å)			Final Pressure
	O	Mn	La					a	b	c	
450	−428.03	−639.99	−855.83	Initial: 58.41 Final: 61.69	−2821.51	−2.8215	500	Initial: 3.88 Final: 3.95	Initial: 3.88 Final: 3.95	Initial: 3.88 Final: 3.95	0.0892
650	−428.09	−640.01	−855.85	Initial: 58.41 Final: 61.72	−2821.69	−2.8217	500	Initial: 3.88 Final: 3.95	Initial: 3.88 Final: 3.95	Initial: 3.88 Final: 3.95	0.0730
800	−428.10	−640.02	−855.85	Initial: 58.41 Final: 61.84	−2821.73	−2.8217	500	Initial: 3.95 Final: 3.95	Initial: 3.95 Final: 3.95	Initial: 3.95 Final: 3.95	0.0852

**Table 7:** Data of theoretical calculations using the CASTEP module with the R$\bar{3}$c space group.

Cut-Off (eV)	Total Energy (eV)			Cell Vol (Å^3^)	Final Energy (eV)	BFGS Final Enthalpy (×10^4^ eV)	BFGS Bulk Modulus (GPa)	Lattice Parameters (Å)			Final Pressure
	O	Mn	La					a	b	c	
450	−430.67	−612.74	−967.81	Initial: 354.03 Final: 365.05	−17504.76	−1.7505	179.37	Initial: 5.53 Final: 5.55	Initial: 5.53 Final: 5.55	Initial: 13.4 Final: 13.39	−0.0160
650	−431.67	−612.76	−967.82	Initial: 354.03 Final: 353.72	−17515.00	−1.7515	257.74	Initial: 5.53 Final: 5.55	Initial: 5.53 Final: 5.55	Initial: 13.4 Final: 13.28	0.0053
800	−431.75	−612.84	−970.07	Initial: 354.03 Final: 353.20	−17514.99	−1.7515	76.78	Initial: 5.53 Final: 5.54	Initial: 5.53 Final: 5.54	Initial: 13.4 Final: 13.28	0.0614

## Data Availability

Data is contained within the article and the Supplementary Materials.

## Supplementary Materials

The following supporting information can be downloaded at: https://www.mdpi.com/article/10.3390/ma16031274/s1, Figure S1: Schematic of the 3D structure of cubic and rhombohedral LaMnO_3_ used for DFT calculations; Figure S2: TEM images and particle size histograms of LaMnO_3_ calcined at 800 °C (A) and 1000 °C (B); Table S1: Comparing the cubic and rhombohedral symmetry structural parameters of Rietveld refinement with theoretical calculation.

## References

[1] Royer S., Duprez D., Can F., Courtois X., Batiot-Dupeyrat C., Laassiri S., Alamdari H. (2014). Perovskites as Substitutes of Noble Metals for Heterogeneous Catalysis: Dream or Reality. Chem. Rev..

[2] Parravano G. (1953). Catalytic Activity of Lanthanum and Strontium Manganite. J. Am. Chem. Soc..

[3] Ciambelli P., Cimino S., De Rossi S., Lisi L., Minelli G., Porta P., Russo G. (2001). AFeO_3_ (A = La, Nd, Sm) and LaFe_1−x_Mg_x_O_3_ perovskites as methane combustion and CO oxidation catalysts: Structural, redox and catalytic properties. Appl. Catal. B Environ..

[4] Arandiyan H., Mofarah S.S., Sorrell C.C., Doustkhah E., Sajjadi B., Hao D., Wang Y., Sun H., Ni B.J., Rezaei M. (2021). Defect engineering of oxide perovskites for catalysis and energy storage: Synthesis of chemistry and materials science. Chem. Soc. Rev..

[5] Alonso J.A., Martínez-Lope M.J., Casais M.T., Fernández-Díaz M.T. (2000). Evolution of the Jahn−Teller Distortion of MnO_6_ Octahedra in RMnO_3_ Perovskites (R = Pr, Nd, Dy, Tb, Ho, Er, Y): A Neutron Diffraction Study. Inorg. Chem..

[6] Wang X., Huang K., Yuan L., Xi S., Yan W., Geng Z., Cong Y., Sun Y., Tan H., Wu X. (2018). Activation of Surface Oxygen Sites in a Cobalt-Based Perovskite Model Catalyst for CO Oxidation. J. Phys. Chem. Lett..

[7] Najjar H., Batis H. (2016). Development of Mn-based perovskite materials: Chemical structure and applications. Catal. Rev..

[8] Tofield B.C., Scott W.R. (1974). Oxidative nonstoichiometry in perovskites, an experimental survey; the defect structure of an oxidized lanthanum manganite by powder neutron diffraction. J. Solid State Chem..

[9] Angel S., Tapia J.D., Gallego J., Hagemann U., Wiggers H. (2021). Spray-Flame Synthesis of LaMnO_3+δ_ Nanoparticles for Selective CO Oxidation (SELOX). Energy Fuels.

[10] Valderrama G., Kiennemann A., de Navarro C.U., Goldwasser M.R. (2018). LaNi_1−x_Mn_x_O_3_ perovskite-type oxides as catalysts precursors for dry reforming of methane. Appl. Catal. A Gen..

[11] Yamazoe N., Teraoka Y. (1990). Oxidation catalysis of perovskites---relationships to bulk structure and composition (valency, defect, etc.). Catal. Today.

[12] Kulandaivelu P., Sakthipandi K., Senthil Kumar P., Rajendran V. (2013). Mechanical properties of bulk and nanostructured La_0.61_Sr_0.39_MnO_3_ perovskite manganite materials. J. Phys. Chem. Solids.

[13] Zhong W., Chen W., Ding W.P., Zhang N., Hu A., Du Y.W., Yan Q.J. (1999). Synthesis, structure and magnetic entropy change of polycrystalline La_1−x_K_x_MnO_3+δ_. J. Magn. Magn. Mater..

[14] Iliev M.N., Abrashev M.V., Lee H.G., Popov V.N., Sun Y.Y., Thomsen C., Meng R.L., Chu C.W. (1998). Raman spectroscopy of orthorhombic perovskite-like YMnO_3_ and LaMnO_3_. Phys. Rev. B.

[15] Rodríguez-Carvajal J., Hennion M., Moussa F., Moudden A.H., Pinsard L., Revcolevschi A. (1998). Neutron-diffraction study of the Jahn-Teller transition in stoichiometric LaMnO_3_. Phys. Rev. B.

[16] Taboada-Moreno C.A., Sánchez-De Jesús F., Pedro-García F., Cortés-Escobedo C.A., Betancourt-Cantera J.A., Ramírez-Cardona M., Bolarín-Miró A.M. (2020). Large magnetocaloric effect near to room temperature in Sr doped La_0.7_Ca_0.3_MnO_3_. J. Magn. Magn. Mater..

[17] Megaw H.D., Darlington C.N.W. (1975). Geometrical and structural relations in the rhombohedral perovskites. Acta Crystallogr. Sect. A.

[18] Wang G., Xu T., Wen S., Pan M. (2015). Structure-dependent electrocatalytic activity of La_1−x_Sr_x_MnO_3_ for oxygen reduction reaction. Sci. China Chem..

[19] Ashok A., Kumar A., Ponraj J., Mansour S.A., Tarlochan F. (2021). Enhancing the electrocatalytic properties of LaMnO_3_ by tuning surface oxygen deficiency through salt assisted combustion synthesis. Catal. Today.

[20] Norby P., Andersen I.G.K., Andersen E.K., Andersen N.H. (1995). The crystal structure of lanthanum manganate (III), LaMnO_3_, at room temperature and at 1273 K under N_2_. J. Solid State Chem..

[21] Qiu X., Proffen T., Mitchell J.F., Billinge S.J.L. (2005). Orbital Correlations in the Pseudocubic O and Rhombohedral R Phases of LaMnO_3_. Phys. Rev. Lett..

[22] Megarajan S.K., Rayalu S., Nishibori M., Teraoka Y., Labhsetwar N. (2015). Effects of Surface and Bulk Silver on PrMnO_3+δ_ Perovskite for CO and Soot Oxidation: Experimental Evidence for the Chemical State of Silver. ACS Catal..

[23] Sardar K., Lees M.R., Kashtiban R.J., Sloan J., Walton R.I. (2011). Direct Hydrothermal Synthesis and Physical Properties of Rare-Earth and Yttrium Orthochromite Perovskites. Chem. Mater..

[24] Yuan X., Meng L., Zheng C., Zhao H. (2021). Deep Insight into the Mechanism of Catalytic Combustion of CO and CH_4_ over SrTi_1−x_B_x_O_3_ (B = Co, Fe, Mn, Ni, and Cu) Perovskite via Flame Spray Pyrolysis. ACS Appl. Mater. Interfaces.

[25] Kaliaguine S., Van Neste A., Szabo V., Gallot J.E., Bassir M., Muzychuk R. (2001). Perovskite-type oxides synthesized by reactive grinding: Part I. Preparation and characterization. Appl. Catal. A Gen..

[26] Acharya S., Mondal J., Ghosh S., Roy S.K., Chakrabarti P.K. (2010). Multiferroic behavior of lanthanum orthoferrite (LaFeO_3_). Mater. Lett..

[27] Li F., Yu X., Chen L., Pan H., Xin X. (2002). Solid-State Synthesis of LaCoO_3_ Perovskite Nanocrystals. J. Am. Ceram. Soc..

[28] Ansari A.A., Ahmad N., Alam M., Adil S.F., Ramay S.M., Albadri A., Ahmad A., Al-Enizi A.M., Alrayes B.F., Assal M.E. (2019). Physico-chemical properties and catalytic activity of the sol-gel prepared Ce-ion doped LaMnO_3_ perovskites. Sci. Rep..

[29] Li Y., Xue L., Fan L., Yan Y. (2009). The effect of citric acid to metal nitrates molar ratio on sol–gel combustion synthesis of nanocrystalline LaMnO_3_ powders. J. Alloys Compd..

[30] Töpfer J., Goodenough J.B. (1997). LaMnO_3+δ_ Revisited. J. Solid State Chem..

[31] Nilsen O., Rauwel E., Fjellvåg H., Kjekshus A. (2007). Growth of La_1−x_Ca_x_MnO_3_ thin films by atomic layer deposition. J. Mater. Chem..

[32] Berger D., Matei C., Papa F., Macovei D., Fruth V., Deloume J.P. (2007). Pure and doped lanthanum manganites obtained by combustion method. J. Eur. Ceram. Soc..

[33] Abdallah H.M.I., Moyo T. (2014). Superparamagnetic behavior of Mn_x_Ni_1−x_Fe_2_O_4_ spinel nanoferrites. J. Magn. Magn. Mater..

[34] Masina P., Moyo T., Abdallah H.M.I. (2015). Synthesis, structural and magnetic properties of Zn_x_Mg_1−x_Fe_2_O_4_ nanoferrites. J. Magn. Magn. Mater..

[35] Tomaszewski P.E., Miniajluk N., Zawadzki M., Trawczyński J. (2019). X-ray study of structural phase transitions in nanocrystalline LaMnO_3+δ_ perovskite. Phase Transit..

[36] Dlamini W.B., Msomi J.Z., Moyo T. (2015). XRD, Mössbauer and magnetic properties of Mg_x_Co_1−x_Fe_2_O_4_ nanoferrites. J. Magn. Magn. Mater..

[37] Zhang L., Zhou Q., He Q., He T. (2010). Double-perovskites A_2_FeMoO_6−δ_ (A = Ca, Sr, Ba) as anodes for solid oxide fuel cells. J. Power Sources.

[38] Mefford J.T., Hardin W.G., Dai S., Johnston K.P., Stevenson K.J. (2014). Anion charge storage through oxygen intercalation in LaMnO_3_ perovskite pseudocapacitor electrodes. Nat. Mater..

[39] Hammami R., Harrouch Batis N., Batis H., Minot C. (2009). Cation-deficient lanthanum manganite oxides: Experimental and theoretical studies. Solid State Sci..

[40] Huang Q., Santoro A., Lynn J.W., Erwin R.W., Borchers J.A., Peng J.L., Greene R.L. (1997). Structure and magnetic order in undoped lanthanum manganite. Phys. Rev. B.

[41] Wei Z., Xia T., Ma J., Feng W., Dai J., Wang Q., Yan P. (2007). Investigation of the lattice expansion for Ni nanoparticles. Mater. Charact..

[42] Ponce S., Peña M.A., Fierro J.L.G. (2000). Surface properties and catalytic performance in methane combustion of Sr-substituted lanthanum manganites. Appl. Catal. B Environ..

[43] Nagabhushana B.M., Chakradhar R.P.S., Ramesh K.P., Shivakumara C., Chandrappa G.T. (2007). Combustion synthesis, characterization and metal–insulator transition studies of nanocrystalline La_1−x_Ca_x_MnO_3_ (0.0 ≤ x ≤ 0.5). Mater. Chem. Phys..

[44] Sui Z.-J., Vradman L., Reizner I., Landau M.V., Herskowitz M. (2011). Effect of preparation method and particle size on LaMnO_3_ performance in butane oxidation. Catal. Commun..

[45] Ortiz-Quiñonez J.-L., García-González L., Cancino-Gordillo F.E., Pal U. (2020). Particle dispersion and lattice distortion induced magnetic behavior of La_1−x_Sr_x_MnO_3_ perovskite nanoparticles grown by salt-assisted solid-state synthesis. Mater. Chem. Phys..

[46] Symianakis E., Malko D., Ahmad E., Mamede A.-S., Paul J.-F., Harrison N., Kucernak A. (2015). Electrochemical Characterization and Quantified Surface Termination Obtained by Low Energy Ion Scattering and X-ray Photoelectron Spectroscopy of Orthorhombic and Rhombohedral LaMnO_3_ Powders. J. Phys. Chem. C.

[47] Tran T.H., Bach T.C., Pham N.H., Nguyen Q.H., Sai C.D., Nguyen H.N., Nguyen V.T., Nguyen T.T., Ho K.H., Doan Q.K. (2019). Phase transition of LaMnO_3_ nanoparticles prepared by microwave assisted combustion method. Mater. Sci. Semicond. Process..

[48] Mahmood A., Warsi M.F., Ashiq M.N., Sher M. (2012). Improvements in electrical and dielectric properties of substituted multiferroic LaMnO_3_ based nanostructures synthesized by co-precipitation method. Mater. Res. Bull..

[49] Coşkun M., Polat Ö., Coşkun F.M., Durmuş Z., Çağlar M., Turut A. (2019). Effect of Os doping on electrical properties of YMnO_3_ multiferroic perovskite-oxide compounds. Mater. Sci. Semicond. Process..

[50] Koriba I., Lagoun B., Guibadj A., Belhadj S., Ameur A., Cheriet A. (2021). Structural, electronic, magnetic and mechanical properties of three LaMnO_3_ phases: Theoretical investigations. Comput. Condens. Matter.

[51] Moreno L.C., Valencia J.S., Landínez Téllez D.A., Arbey Rodríguez M.J., Martínez M.L., Roa-Rojas J., Fajardo F. (2008). Preparation and structural study of LaMnO_3_ magnetic material. J. Magn. Magn. Mater..

[52] Iniama G., de la Presa P., Alonso J.M., Multigner M., Ita B.I., Cortés-Gil R., Ruiz-González M.L., Hernando A., Gonzalez-Calbet J.M. (2014). Unexpected ferromagnetic ordering enhancement with crystallite size growth observed in La_0.5_Ca_0.5_MnO_3_ nanoparticles. J. Appl. Phys..

[53] Sarkar T., Ghosh B., Raychaudhuri A.K., Chatterji T. (2008). Crystal structure and physical properties of half-doped manganite nanocrystals of less than 100-nm size. Phys. Rev. B.

