# Supplementary material for: Phase Transition of High-Surface-Area Glycol–Thermal Synthesized Lanthanum Manganite
Source: Materials (Basel). 2023 Feb 2;16(3):1274. doi: 10.3390/ma16031274 (PMC9920577; doi:10.3390/ma16031274)
Supplement: Supplementary file 1 [file materials-16-01274-s001.zip › materials-2076054-supplementary.pdf]

Phase Transition of High-Surface-Area Glycol–Thermal Synthesized Lanthanum Manganite

Victor O. Anyanwu, Holger B. Friedrich, Abdul S. Mahomed, Sooboo Singh and Thomas Moyo *

School of Chemistry and Physics, Westville Campus, University of KwaZulu-Natal, Durban 4000, South Africa
* Correspondence: moyo@ukzn.ac.za; Tel.: +27-31-2608331

Supplementary information

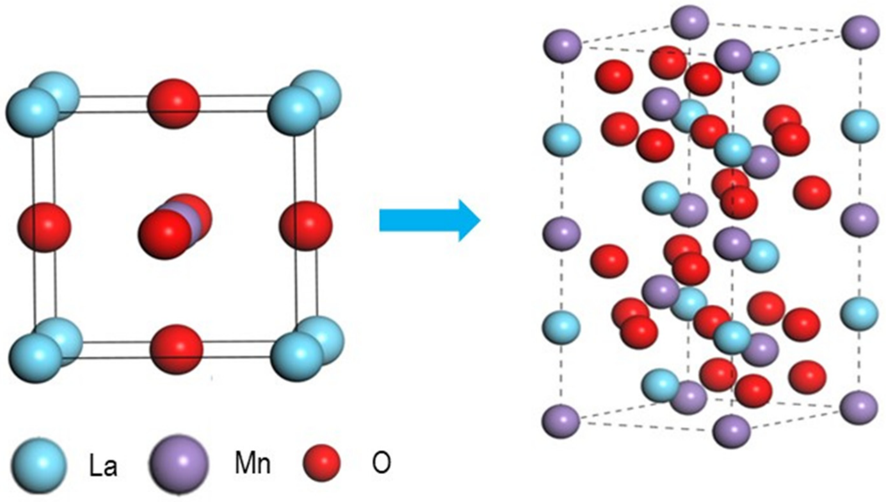

Figure S1 Schematic of the 3D structure of cubic and rhombohedral $LaMnO_3$ used for DFT calculations.

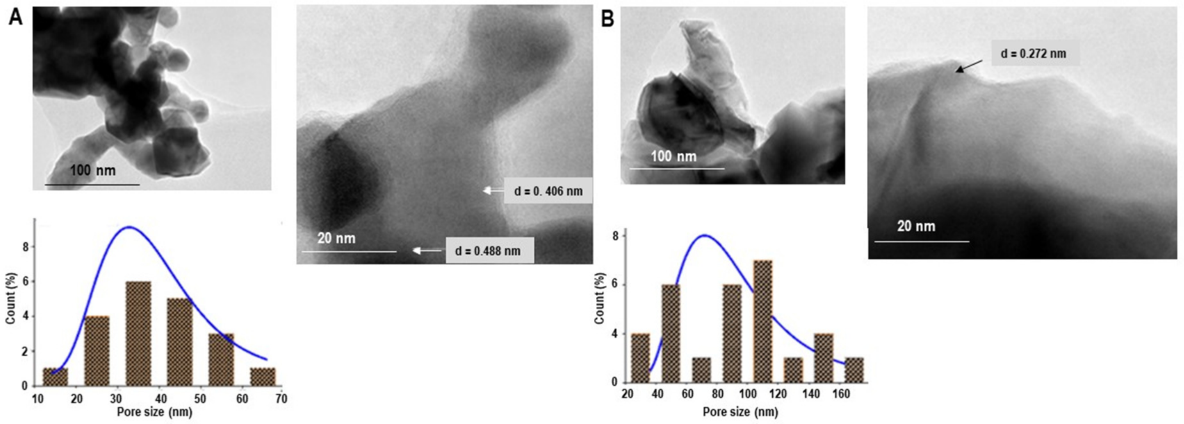

Figure S2 TEM images and particle size histograms of $LaMnO_3$ calcined at 800°C (A) and 1000°C (B).

Table S1 Comparing the cubic and rhombohedral symmetry structural parameters of Rietveld refinement with theoretical calculation.

	Experimental				Theoretical			
Phase	Lattice parameters (Å)			Cell volume ($Å^3$)	Sample	Lattice parameters (Å)			Cell volume ($Å^3$)
	a	b	c			a	b	c	
LM 700	Initial:3.88	Initial:3.88	Initial:3.88	Initial: 58.41	Pm-3m	Initial:3.88	Initial:3.88	Initial:3.88	Initial:58.41
	Final:3.88	Final:3.88	Final:3.88	Final: 58.46		Final:3.95	Final:3.95	Final:3.95	Final:61.69
LM 1000	Initial:5.53	Initial:5.53	Initial:13.4	Initial:354.03	$R\bar{3}c$	Initial:5.53	Initial:5.53	Initial:13.4	Initial:354.03
	Final:5.52	Final:5.52	Final:13.36	Final: 353.00		Final:5.54	Final:5.54	Final:13.28	Final:353.20
